# Experience of care of hospitalized newborns and young children and their parents: A scoping review

**DOI:** 10.1371/journal.pone.0272912

**Published:** 2022-08-29

**Authors:** Charity Ndwiga, Charlotte Elizabeth Warren, Chantalle Okondo, Timothy Abuya, Pooja Sripad

**Affiliations:** 1 Population Council, Nairobi, Kenya; 2 Population Council, Washington, DC, United States of America; Public Library of Science, UNITED KINGDOM

## Abstract

**Introduction:**

Several global initiatives put parent involvement at the forefront of enabling children’s well-being and development and to promote quality of care for newborns and hospitalized young children aged 0–24 months. Scanty evidence on mistreatment such as delays or neglect and poor pain management among newborns exists, with even less exploring the experience of their parents and their hospitalized young children. To address this gap, authors reviewed research on experience of care for hospitalized young children and their parents, and potential interventions that may promote positive experience of care.

**Methods:**

A scoping review of English language articles, guidelines, and reports that addressed the experiences of care for newborns and sick young children 0–24 months in health facilities was conducted. Multiple databases: PubMed, PROSPERO, COCHRANE Library and Google Scholar were included and yielded 7,784 articles. Documents published between 2009 and November 2020, in English and with evidence on interventions that addressed family involvement and partnership in care for their sick children were included.

**Results:**

The scoping review includes 68 documents across 31 countries after exclusion. Mistreatment of newborns comprises physical abuse, verbal abuse, stigma and discrimination, failure to meet professional standards, poor rapport between providers and patients, poor legal accountability, and poor bereavement and posthumous care. No literature was identified describing mistreatment of hospitalized children aged 60 days– 24 months. Key drivers of mistreatment include under-resourced health systems and poor provider attitudes. Positive experience of care was reported in contexts of good parent-provider communication. Three possible interventions on positive experience of care for hospitalized young children (0–24 months) emerged: 1) nurturing care; 2) family centered care and 3) provider and parental engagement. Communication and counseling, effective provider-parental engagement, and supportive work environments were associated with reduced anxiety and stress for parents and hospitalized young children. Few interventions focused on addressing providers’ underlying attitudes and biases that influence provider behaviors, and how they affect engaging with parents.

**Conclusion:**

Limited evidence on manifestations of mistreatment, lack of respectful care, drivers of poor experience and interventions that may mitigate poor experience of care for hospitalized young children 0–24 months especially in low resource settings exists. Design and testing appropriate models that enhance socio-behavioral dimensions of care experience and promote provider-family engagement in hospitals are required.

## Introduction

Every year, millions of children under 5 years of age die worldwide, mostly from preventable causes, including preterm birth, neonatal sepsis, birth asphyxia and defects, pneumonia, diarrhea, malaria, and nutrition-related conditions. The United Nations’ Sustainable Development Goals include targets the reduction of neonatal mortality to 12 deaths per 1,000 live births and under five mortality to 25 deaths per 1,000 live births by 2030 [1, 2]. Despite the substantial global progress in reducing child mortality over the past few decades, a child’s ability to survive and thrive remains an urgent concern. The Every Newborn Action Plan (ENAP) initiative aims to have: *“a world in which there are no preventable deaths of newborns or stillbirths*, *where every pregnancy is wanted*, *every birth is celebrated*, *and women*, *babies and children survive*, *thrive and reach their full potential”* [3–5]. ENAP identifies the role of parents, families, and communities to enable children’s well-being and development and promote quality care. It places parental and family involvement at the forefront of provision of care for newborns and young children and emphasizes the importance of engaging men as caregivers and decision-makers in maternal and newborn care-seeking behavior [3].

The quality of care for hospitalized newborns and young children is intrinsically linked to experience of care across the life-course continuum (starting from their mothers’ pregnancy through her childbirth experience and thereafter) and recognized in global frameworks that view quality as a combination of service delivery and experience of patient care [4–6]. Quality of newborn and pediatric care are further articulated by the World Health Organization (WHO)’s maternal and newborn health (MNH) care and pediatric quality of care frameworks and eight standards for quality of pediatric care as well as the standards for improving quality of care for small and sick newborns in health facilities [5, 7, 8]. These quality domains recognize children’s health as encompassing a range of distinct physical, psychosocial, developmental and communication needs [6]. Specifically, positive experience of care entails meaningful family participation in their children’s care, respect, protection and fulfillment of children’s rights and emotional and psychological support. While these eight domains offer quality aspirations in principle, there is less known about their realization in practice. Positive experience of care for sick children can reduce parental anxiety and improve communication between parents/caregivers and health professionals [9]. However, although a growing body of evidence around experience of care including mistreatment of women during childbirth exists [10–14], there has been less attention to understand the experience of parents of newborns and young children up to 24 months. There have been few concerted effort to identify or address manifestations of mistreatment of newborns and hospitalized young children [15]. Sacks found that mistreatment in newborns include failure to meet a professional standard of care, stigma and discrimination, and health system constraints, delays or neglect, non-consented, physical (poor pain management or rough handling and verbal abuse, lack of communication with parents’ lack of legal accountability (recognition for the newborn as a person) and bereavement care [15]. Similarly, there is little evidence on interventions that may mitigate poor experience of care and promote positive care experiences among newborns and young children. As a result, appropriate models that enhance the socio-behavioral dimensions of care experience and promote family-engagement are limited in low income settings [16].

To fill this gap, the authors conducted a scoping review to map the research on experience of care and related interventions for young children from birth to 24 months and their parents in hospitalized and outpatient settings. The focus was on manifestations of mistreatment and their drivers during care for young children up to 24 months, positive and negative experiences of care and, potential interventions that reduce mistreatment, promote provider communication and family engagement in their children’s care globally, including in low- and middle-income countries. Evidence from this scoping review alongside formative research in select hospitals in Kenya (not presented in this paper) will be used to identify a contextually relevant model to improve experience of care for parents and families seeking services for hospitalized newborns, infants and young children up to 24 months in a low-income setting. For the purpose of this paper, we use the term “young children” for those who are 0 to 24 months unless otherwise specified.

## Methods

Scoping reviews map key concepts within a research area, the main sources and types of evidence available, and can be used for concurrent pragmatic aims, including summarizing and describing a particular concept (e.g. experience of care for young children and their parents or related-interventions), its characteristics, and identification of gaps and areas of future research and practice [17, 18]. Given our study is embedded in a broader implementation science approach to inform the development of a theory of change framework, we applied a scoping review as a methodological approach to help the authors map and synthesize relevant studies on experience of care and mistreatment for sick young children 0–24 months, interventions on family involvement in caring for sick children. This approach was conducive to eliciting thematic gaps and programmatic recommendations for subsequent studies and interventions to improve experience of care, reduce mistreatment and encourage family engagement and involvement care of young sick children.

### Search strategy

**Fig 1:**
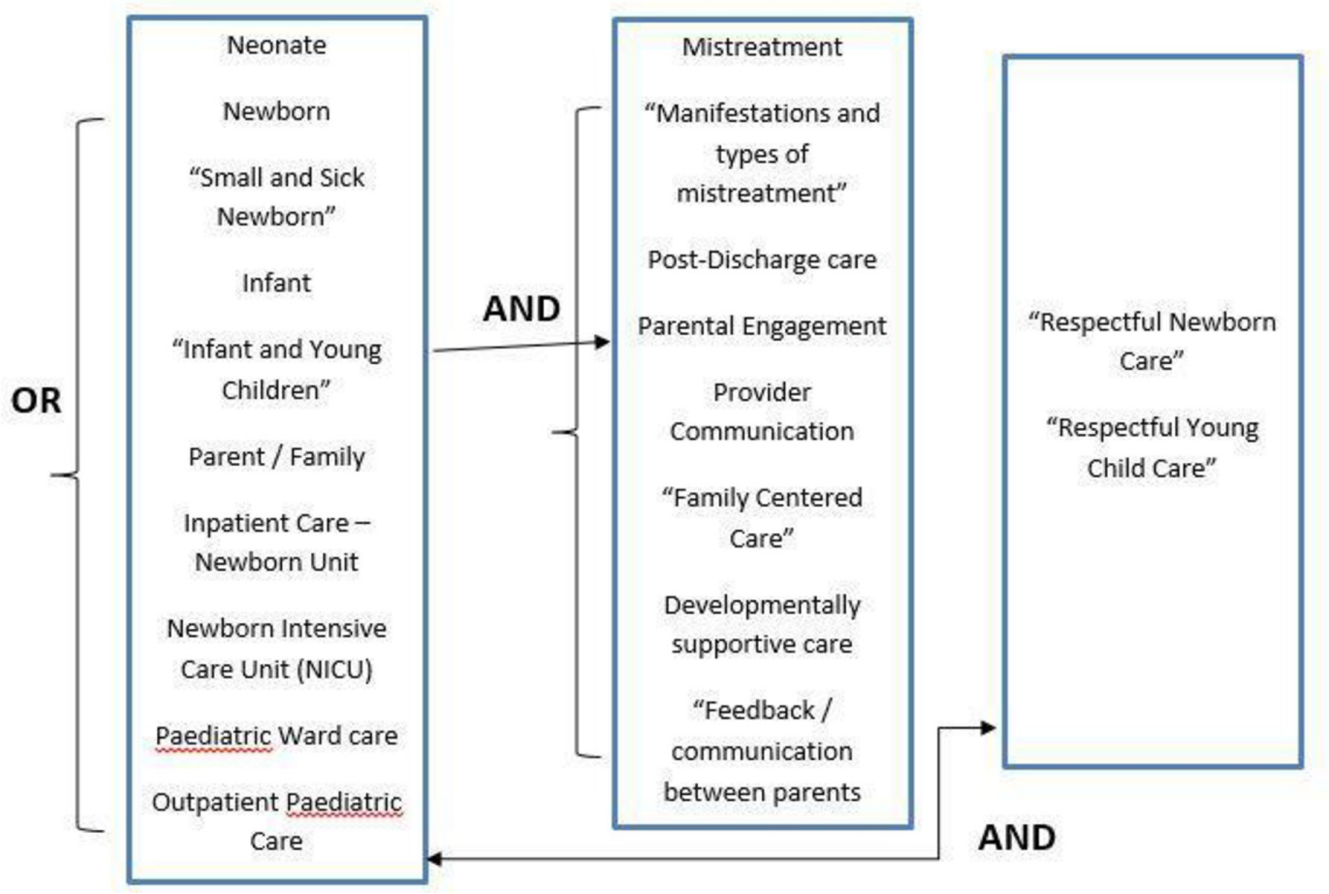
Combination of keywords and Boolean terms used in the search for literature.

Our search strategy drew on guidance from the Preferred Reporting Items for Systematic reviews and Meta-Analyses extension for Scoping Reviews (PRISMA-ScR) [19]. Five reviewers identified the purpose and research question and established a common understanding of relevant terminology to use, identify the core concepts in experience of care and mistreatment, and interventions involving families and parents in the care of sick young children. Three reviewers applied an iterative approach, characteristic of scoping reviews, to select relevant published and grey literature, formal reports, WHO guidelines, standards, and clinical protocols that addressed the experiences of care for newborns and sick young children 0–24 months in health facilities. English language articles, guidelines, and reports were identified by searching multiple databases: PubMed, PROSPERO, COCHRANE Library and Google Scholar. Additional studies were identified through articles shared by experts in newborn and child health and based on reference lists within included articles and reports. All retrieved references were entered in Mendeley Reference Manager Software and duplicates removed. The search terms or key words used are presented in Fig 1 including how the Boolean principles “AND, OR” were applied during the search.

The search terms and their corresponding definitions are presented in S1 Table, some of the definitions were adopted from literature and the recently released report on “*Nurturing Care for Small and Sick Newborns*: *Evidence Review and Country Case Studies”* [20]. Documents were included if they were published between January 2009 and November 2020, published in English and included newborns, infants and young children up to 24 months as well as evidence on interventions that addressed integrated care, nurturing care, quality of care or family-centered care. Qualitative, quantitative, and mixed methods studies were included as well as narrative, literature, and systematic reviews. While literature was drawn globally, a focus was given to the sub-Saharan Africa region by including it in each of the search terms, only 13 out of 68 studies/documents were identified. Documents were excluded if they only provided evidence on children older than 24 months, not available in English, published before 2009 or were not relevant to experience of care.

### Data extraction

Two reviewers (CN and CO) extracted data by reading the full text articles, guidelines and reports that met the eligibility criteria and retained through the iterative charting process. Reviewers extracted information, using Microsoft Excel, on each document’s publication-related data (authors, title, reference), geography and context, study and data type, manifestations and types of mistreatment and positive experiences of newborns and sick young children, of family centered care (FCC), quality of care, intervention elements—including partners and funders. They reviewed the relevance and type of evidence and summarized these broad emerging themes to map out areas for future programmatic research.

## Results

### General overview

The initial search in PubMed, PROSPERO, COCHRANE Library and Google Scholar yielded 7,784 results and after exclusions, 246 were retrieved as potentially eligible papers.

**Fig 2:**
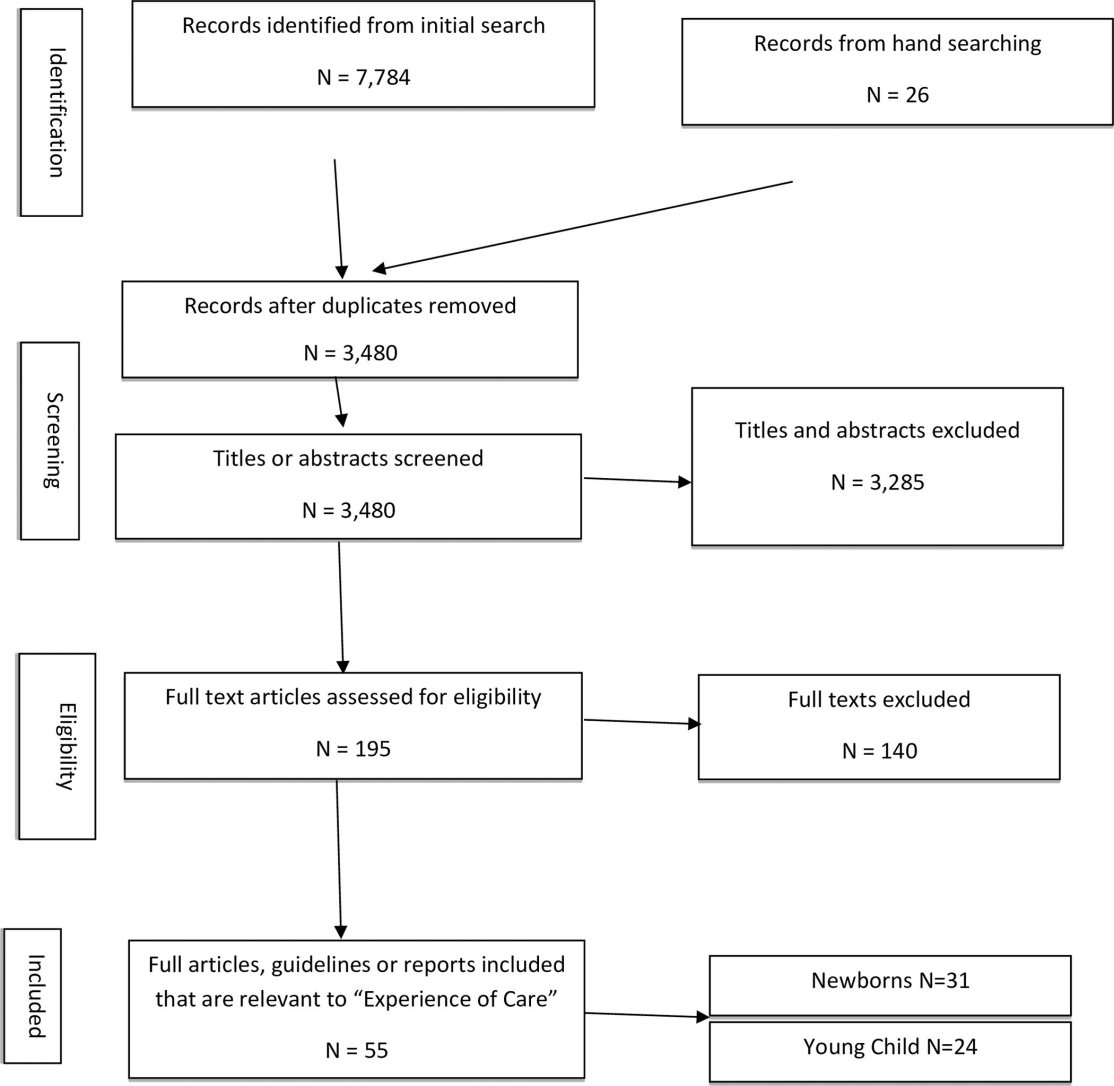
Flow diagram of search and study inclusion process.

Fig 2 illustrates the search strategy.

After exclusions, 68 published papers, grey literature, guidelines and strategy documents aimed at defining normative practices among young children 0–24 months of age were included. The analysis synthesized findings from single- and multi-country studies conducted across 31 countries, including Global (n = 18); high income countries in Europe (n = 13), North America (n = 15) and Australia (n = 2); low- and middle-income countries (LMICs) in South America (n = 2); Asia (n = 6) and sub-Saharan Africa (n = 13). Many papers focused on newborns aged between 0–28 days including stillbirths, premature babies, and neonatal deaths (n = 42). The remaining literature centered on children 59 days and older (n = 26); of these, very few papers had evidence that was exclusive to young children up to 24 months old (n = 4). The remaining 19 (of the 26) addressed under five or pediatric care in older children but were retained as they met the other inclusion criteria and featured learnings on interventions around the 0-24-month age group.

The results from the scoping review are described under three main domains: 1) experience of care for hospitalized young children and their caregivers; 2) drivers of negative experience of care of hospitalized young children and their caregivers; and 3) interventions to promote positive experience of caregivers and their hospitalized young children. Where possible, each domain describes issues pertaining to hospitalized newborns and young infants (0–59 days) and infant/young child (60 days– 24 months) separately. We found 15 articles on experience of care (Table 1), 11 on drivers of poor experience of care (Table 2), and 32 on interventions that may reduce negative experiences of care (Table 3).

**Table 1:** Summary of literature on experiences of care/mistreatment.

No	Authors	Study Design	Geographic Location	Age Group	Purpose	Findings
1.	Sacks (2017)	Literature review	Global	0–59 days	Using 7 categories previously developed for respectful maternity care generally, a literature review was conducted on mistreatment of newborns.	The review revealed examples of mistreatment of newborns: failure to meet a professional standard of care, stigma and discrimination, and health system constraints. Many instances of mistreatment of newborns related to neglect and non-consented care rather than outright physical or verbal abuse. Two additional categories: legal accountability and poor bereavement care.
2.	Altimier and Phillips (2016)	Mixed methods, review of evidence	Global	0–59 days	Training and consultative process—based quality improvement designed to optimize the NICU environment and caregiving practices in order to facilitate the best outcomes for premature infants and their families.	Authors describe that infant in the NICU may demonstrate a developmentally unexpected sensory stress response. Exposed to painful, repeated, and unpredictable medical procedures, and to physical pain or discomfort related to illness, these infants may not have consistent support from a parent or professional caregiver to provide a buffer to help them stay regulated and recover from these stresses. Parents also lack autonomy in making decision about care of their children.
3.	Costello, A (2017)	Editorial review	India	0–59 days	Case studies of the effect of period of separation between mother and infant, in NICU, that might be traumatic to bonding, and long-term consequences for the mother-baby relationship.	Separation between mother and infant in an ICU might be traumatic to bonding and have long-term consequences for the mother and baby relationship, may affect onset of lactation and impair infant growth. Those with pre-term babies risk not being psychologically prepared for parenthood. Parent of sick infant may feel depressed, intensely anxious and create irrational fears about malformations.
4.	Ellis A; Chebsey C et al (2016)	Mixed- methods systematic review	Europe, North America, Australia and South Africa	Stillbirth	Review to inform research, training and improve care for parents who experience stillbirth.	Parental and staff findings were often related. Parents reported distress caused by midwives hiding behind ‘doing’ and ritualizing guidelines whilst staff described distancing themselves from parents and focusing on tasks as coping strategies.
5.	Forcada-Guex, Borghini (2011)	Quantitative survey, cross sectional study	Switzerland	0–6 months	Explores the links between maternal posttraumatic stress, and maternal attachment representation and the infant and mother–infant dyadic interactions.	Full-term mothers more likely to follow a “Cooperative” dyadic pattern of interaction with the infant and demonstrate balanced representations of the infant. Preterm mothers with high posttraumatic stress symptoms were more likely to follow a “Controlling” dyadic pattern of interaction, with more distorted representations.
6.	Lungu et al., 2016	Qualitative: descriptive and exploratory	Lilongwe, Malawi’	0–5 years	Explores healthcare-seeking practices for common childhood illnesses focusing on use of biomedical health services and perceived barriers to accessing under-five child health services in urban slums	Long waiting times; late facility opening times; negative attitude of health workers; suboptimal examination of the sick child, cost of services and dehumanizing remarks and actions by providers discouraged care givers from subsequent use of service in health facilities.
7.	Gangi et al (2013)	Quantitative, experimental	Rome, Italy	0–60 days	Describe the causes of PTDS and emotional reaction of parents of premature babies in NICUs	Alteration of parental role and a history of anxiety may lead to development of PTSD in parents with premature neonates. Familiarization with NICU environment and increasing parent participation in their baby’s care during the life improves parental role perception.
8.	Guimarães H, et al. (2015)	Review	Portugal	0–59 days	Review of family involvement in care of sick young infants	The birth of a small sick baby represents a well-known emotional crisis for parents and family. A multidisciplinary approach to the care of newborns in NICUs is essential for child development. Essential to optimize care for immature newborns, as well as the relationship between parents and professionals.
9.	Kuo et al 2012	Commentary	USA	Age not specified. Referred to pediatric care	It highlights the advances in Family Centered Care (FCC) practices in child health and suggest ways to advance the state of FCC in paediatric health care.	FCC principles are best learned through daily exposure and practice. Language should be respectful, care plans should be made jointly, and clinical decisions should consider the context of the family and community. Family presence at bed rounds also to be implemented and evaluated as part of quality improvement.
10.	Ronald and Snelson (2018)	Discussion on decision making in pediatrics	Global	0–5 years–sick children	Highlights physiology, communication, heuristics and external elements as factors which influence decision-making and discusses how incidence of disease and seniority of clinician impact might influence outcomes.	Decision making in pediatrics is influenced by factors dependent on child and on the clinician. Clinicians should use published evidence, guidelines, decision-making tools and available expertise wherever possible to improve their understanding of how to make the best decision in any given clinical scenario. Importance of parents’ involvement in the decision-making process is emphasized.
11.	Tamburlini et al (2011)	Quantitative cross sectional	Albania, Turkmenistan and Kazakhstan	Newborns (NICU) 0–28 days	Assess the quality of maternal and newborn care in three countries, using an innovative approach.	Neonatal care scored better than obstetric care. Lack of information, insufficient support during labour and lack of companionship are main issues. Actions to improve quality of care were identified at facility and central level and framed according to health system functions.
12.	Bazzano et al., 2017;	A comprehensive summary of qualitative data	Low-Income Countries as defined by The World Bank Group Country and Lending Groups	0–2 year	Review related to parental experiences of infant and young child feeding in low-income countries, synthesizing information on the barriers and facilitators that may relate to interventions to impact nutrition, survival, growth and development	An overview relating parental perspectives on infant and child dietary patterns, in the interest of providing insights for developing, improving, and scaling nutrition interventions. E.g. the perception of a lack of breastmilk: including physical signs that milk is absent/insufficient, beliefs about colostrum, and traditional beliefs of when the milk comes in. Delayed initiation of breastfeeding beyond 12 hours often led to pre-lacteal feeding.
13.	Veronez et al (2017)	Qualitative, descriptive and exploratory	Brazil	Premature infants (NICU) 0–59 days	To describe the process of nursing care for mothers during the hospitalization and discharge of premature babies.	1) Experiencing a premature baby forces mothers to face the prospect of having or not having their babies, triggering feelings of helplessness, emotional instability and anxiety; 2) Participating in caring for the child helps to strengthen the bond between mother and baby and where nurses play a crucial role by providing guidance and support; 3) Discharge of the baby: family expectations–anxious to know when the baby will be discharged.
14.	WHO (2018)	Guidelines for improving QoC for young children and adolescents	Global	0–15 years	Second series of standards for improving the quality of care for children (aged 0–15 years) in health facilities	Outlines 8 standards of childcare include: communication with children and families is effective, responds to needs and preferences; children’s rights are protected; children and families receive educational, emotional and psychosocial support that is sensitive to their needs and strengthens their capability and care. Competent and motivated, empathic staff.
15.	Guiller, Cristiana A et al., 2009	Qualitative study	Brazil	0–28 days	To understand the experience of caring for a child with a congenital anomaly from the family’s perspective	Parents of newborns with congenital deformities initially face difficult experiences. These are marked with a process of moments of unbalance, physical and emotional stress and moments of strength, coping and overcoming.
16.	WHO (2020)	Guidelines on protecting, promoting and supporting breastfeeding for small, sick and preterm newborns	Global	0–28 days	Revision based on 2018 WHO guidelines on the baby-friendly hospital initiative for small, sick and preterm newborns	Heath newborn units with noisy, brightly lit, and intimidating and without much privacy would limit breast feeding for small, sick and preterm newborns.
17.	Horwood, C.; et all 2019	Qualitative	Sub-Saharan Africa: South Africa	0–28 days	To explore care of newborn babies admitted to neonatal units in district hospitals	Parents perceived providers as being rude or withholding information or not listening to mothers; non-consented care; speaking loudly about baby’s condition without consideration for privacy and confidentiality. On the other hand, providers’ perspective, they described mothers not following instructions (not washing hands prior to entering neonatal unit); giving incorrect or misleading information. While positive communication was reported by many mothers which led to them feeling empowered and participating actively in the care of their babies, with incidents of poor communication.
18.	Klug J et al., 2019	Quantitative	USA—Delaware	0–12 months	To create and test a bedside visual tool to increase parent partnership in developmentally supportive infant care after cardiac surgery.	Parents were more often observed participating in rounds, asking appropriate questions, providing emotional comfort, assisting with daily care routines and changing diapers. Staff perceived that the tool was generally useful for the patient and the family but was sometimes overlooked or not used. Use of a bedside visual tool may lead to increased parent partnership in care for infants after cardiac surgery
19.	Brodsgaard H, etal., 2019	A qualitative review and meta-synthesis.	Global	0–28 days	To explore how parents and nurses experience partnership in neonatal intensive care units and to identify existing barriers and facilitators to a successful partnership.	Through a meta-aggregative approach, parents reported being respected and listened to, trust and sharing knowledge, and the second synthesis embraced the categories: space to learn with guidance, encouraging and enabling, being in control. In constructing the categories, findings were identified as characteristics, barriers and facilitators to application.
20.	Kasat K’., etal 2020	Quantitative	USA—New York	0–28 days	To implement an “Empathy Workshop” focused on improving Neonatal Intensive Care Unit (NICU) health care provider communication skills.	Families reported the staff team were better at meeting their needs their emotional support and information on NICU support groups; communication skills self-assessment at 6 months post workshop was higher in all questions compared to baseline. NICU medical and nursing providers reported feeling better prepared for interactions with parents—they were more comfortable with daily communication, discussing end of life issues, managing anxiety around difficult conversations, comforting a sad family and handling a combative situation.

**Table 2:** Summary of literature reviewed on drivers of experience of care.

	Authors	Study Design	Geographical Location	Age Group	Purpose	Findings
	Sacks (2017)	Literature review	Global	0–59 days	A literature review on mistreatment of newborns.	Health system structures such as *inadequate providers*, *lack of equipment and running water contribute to mistreatment*.
1	WHO, UNICEF., 2017	Review	Global	Newborns 0–59 days	Review of Every Newborn Action Plan	“The quality of childcare is suboptimal in many high-burden countries. Facilities are poorly equipped, and/or lack lifesaving commodities for women and newborns, including appropriate referral services. *The standard of provider education is often low*, *and staff shortages and low remuneration lead to poor morale and quality of care*.”
3	APHRC (2014)	Review	Kenya, Gambia and Cambodia	0–59 + under 5 years	To draw lessons from successful Baby Friendly Hospital Initiative and Baby Friendly Community Initiative (BFCI) projects with a view of informing similar projects and programs in the country.	Short hospital stays for mothers who birth in a health facility, late initiation in breastfeeding; giving water and fluids to newborns and early complementary feeding (as early as 3 months after birth) are some examples that strongly influenced how mothers fed their infants. *Competent*, *motivated empathetic and trained human resources (For the BFHI and BFCI—health facility and community) was central to the plan to promote exclusive breastfeeding*.
4	Bee, Shiroor and Hill (2018)	Mixed methods systematic review	SSA most studies were from Ethiopia, Ghana, Malawi, Tanzania and Uganda	0–59 days	Reviews quantitative and qualitative data from SAA on the prevalence of key immediate newborn care practices and the factors that influence them	Common beliefs across studies: delayed drying and wrapping of infant because birth attendants focused on the mother; bathing newborns soon after delivery to remove ‘dirt and blood’; negative beliefs about the vernix; applying substances to the cord to make it drop off quickly; and delayed breastfeeding due to perception of a lack of milk or because the baby needs to sleep after delivery or does not show signs of hunger.
5	Callaghan-Koru, J., Seifu A., et al (2013)	Quantitative Retrospective	Ethiopia	0–59 days	Describes newborn care practices reported by recently delivered women (RDWs) in four regions of Ethiopia.	Majority of women had one ANC contact at a health facility, few women had PNC contact with a provider. Practices contrary to WHO recommendations included bathing within the first 24 hours; Butter and other substances applied to the cord. No large differences for most essential newborn care (ENC) indicators between facility and home births.
6	de Graft- Johnston J., et al (2017)	Quantitative Observational study	Sub Saharan Africa	0–59 days Newborns	Presents information on the quality of newborn care services and health facility readiness to provide newborn care in 6 African countries, and to advocate for the improvement of providers’ ENC knowledge and skills.	Major deficiencies exist for ENC supplies and equipment, poor provider knowledge and performance of key routine ENC practices, particularly for immediate skin-to-skin contact and breastfeeding initiation. Of newborns who did not cry at birth, 89% either recovered on their own or through active steps taken by the provider through resuscitation but a third of providers were able to demonstrate ventilation skills correctly.
7	New K et al., (2019)	Evidence synthesis and country case studies	Global	0–28 days Small and sick newborns	To determine the best practices to support FCC to promote nurturing care and early childhood development for small and sick newborns in-facility and post-discharge.	The barriers to nurturing care may include; Service readiness for skin-to-skin, KMC implementation; crowded, noisy units, lack of privacy, uncomfortable beds, and a lack of food and supplies; Lack of facility policies, Inadequate and under-resources health systems, human resources and the environment.
8	Onarheim et al (2017)	Qualitative observational	Ethiopia	0–59 days	To examine family’s decision making and health care seeking for sick newborns in Butajira, Ethiopia	The health of the newborn not always the family’s priority. As newborns were perceived as not yet useful members of the household … and while sickness was recognized as dangerous for the ill newborn, seeking health care could be harmful for the economic survival of the family. Until the baby had survived the first vulnerable weeks and months of life, the unknown newborn was not yet seen as a social person by the community.
9	Shah D and Dwivedi L (2013)	Qualitative, case studies	India	Newborns 0–59 days	To describe deviations from the essential newborn practices followed during hospital and home delivery.	There is less prevalent practice of ENC among all cases irrespective of place of delivery and the health- personnel facilitating delivery. Habitual traditional/ tribal newborn care methods challenge the practice of prescribed ENC
10	MC Avila, et al., 2020	Qualitative	Southwest, Spain	0–28 days	To describe and understand the experiences of parents in relation to professional and social support following stillbirth and neonatal death.	Grieving parents reported lack of continued care/support for despite sustained access to postnatal support (mental, social). Parents were particularly feeling a sense of loneliness in their experience of perinatal death—at hospital and socially (after) which compounded negative experience of loss of a child. This loss and difficulty was worse for parents who had no other children to distract them (a coping mechanism to process grief)
11	Baughcum AE, et al., 2020	Quantitative	United States, Ohio	0–28 days	To examine parents’ perceptions of their infant’s End of Life experience (e.g., symptom burden and suffering) and satisfaction with care in the NICU	Mothers felt they did not fully understand the cause of death/medical aspects. They weren’t completely satisfied with health staff assistance in EOI decision-making. They were also slightly less satisfied with overall care than fathers. Both parents had low satisfaction scores with their emotional needs being met.

**Table 3:** Summary of literature on interventions implemented.

No	Authors	Geographic Location	Methods	Age Group	Intervention	Outcomes of measurements	How interventions were implemented	Findings
**Nurturing care**								
1.	Altimier and Phillips (2016)	Global	Mixed methods, review of evidence	0–59 days	The Neonatal Integrative Developmental Care Model on Nurturing care.	The seven neuroprotective core measures are depicted as overlapping petals of a lotus 1) Healing Environment, 2) Partnering with Families, 3) Positioning & Handling, 4) Safeguarding Sleep, 5) Minimizing Stress and Pain, 6) Protecting Skin, and 7) Optimizing Nutrition. Skin to Skin Contact (SSC) is considered the foundation for care of infants in the NICU and its importance as the normal environment and the ideal place of care are described	eLearning, didactic education, hands-on interactive workshops, physician sessions, and in-unit consultation to all individuals who care for premature infants in a NICU to optimize the NICU environment and caregiving practices in order to facilitate the best outcomes for premature infants and their families.	Intervention found to improve noise and light levels in the NICU, improve infant medical outcomes, improve staff satisfaction, improve family satisfaction, decrease length of stay (LOS) and hospital costs in NICU.
2.	New K, et al., 2019	Colombia, India, Nepal, Philippines, Rwanda, Sweden, and the United States	A review of evidence	0–59 days	Nurturing care approaches & FCC for in-facility small and sick newborns and post discharge care at the community level.	Interventions include; Skin-to-skin/kangaroo care, Nutrition (breastmilk feeding and breastfeeding), Sensory environment, Stress and pain, Supportive positioning, Protecting and promoting sleep, Protecting skin Age-appropriate stimulation and interactions, Partnering with parents/families, follow-up and screening specifically in relation to neurodevelopment and Laws and policies	Many individual and models of care interventions include adaptation or revision of policies, services and infrastructure support to create nurturing care environments, inpatient and, post-discharge education. Family-centered care environment, improved knowledge, improved family satisfaction, decision making and healthcare action. Developmentally supportive care environment—staff attitudes, beliefs, interpersonal skills, Quality Improvement,—provider competence supportive supervision and mentoring.	The interventions improved exclusive breastmilk feeding as soon as medically able for small and sick newborns unable to breastfeed—early initiation, exclusive breastfeeding for all newborns who can suckle—continued breastfeeding after 6 months. However, assessment and management of pain is still poorly undertaken with reports that pain management (pharmacological or non-pharmacological) only undertaken in about 50% of the time for painful procedures in neonatal unit. NICU infrastructure for warmth lighting was limited.
3.	World Health Organization 2018	Global	None	0–3 years	The new Nurturing	None–not a study	The framework draws on state of art evidence state-of-the-art evidence on how early childhood development unfolds to set out the most effective policies and services that will help parents and caregivers provide nurturing care for babies. It recommends training of care givers and parents and partnership in care.	It opines that nurturing care starts before birth, when mothers and other caregivers can start talking and singing to the fetus. By the end of second trimester of pregnancy, the growing fetus can hear. And, from birth, the baby can recognize the mother’s voice. Early bonding is facilitated by skin-to-skin contact, breastfeeding and the presence of a companion to support the mother. These also build the foundations for optimal nutrition, quality interactions and care thereafter.
1.	Britto PR et al., 2017	Global	A review	0–60 months	Nurturing care: promoting early childhood development.	To provide a comprehensive analysis of early childhood development interventions across the five sectors of health, nutrition, education, child protection, and social protection. One of the interventions reviewed is nurturing care.	Through combining sectoral interventions such as the Care for Child Development Program, delivered by Lady Health workers in Pakistan with elements of nurturing care and protection to improve child outcomes. Likewise, nurturing care and protection can be combined with interventions that offer parenting support and skills.	The review finds that Interventions that integrate nurturing care and protection can target multiple risks to developmental potential at appropriate times and can be integrated within existing preventive and promotive packages.
**Family Centered Care**								
	New K, et al., 2019	Colombia, India, Nepal, Philippines, Rwanda, Sweden, and the United States	A review of evidence	0–28 days	Nurturing care approaches & FCC for in-facility small and sick newborns and post discharge care at the community level.	Key aspects of family-centered care (FCC) such as communication, collaboration, respect, and flexible, culturally competent and responsive caregiving.	Family involvement in decision making and communication (use of simple language by clinicians).	Engaging parents early with good communication, education, participation in care giving and decision-making benefits short term outcomes for newborns such as breastfeeding, growth, readiness for discharge, distress, and stress; and for parents: reduced stress, increased confidence and positive parent-infant interactions.
1.	Champlain Maternal Newborn Regional Program (CMNRP), 2015	Canada	A framework of evidence	0–28 days	The framework serves to simplify and organize the vast FCC literature to facilitate easier implementation and to support healthcare organizations.	Aspects of FCC namely dignity and respect, information sharing collaboration and participation are described.	Healthcare providers partner with families as they share the same goals: safe, high quality, and satisfying care with the best possible outcomes.	Partnerships with families is about how to put families first, not only with individual healthcare providers, but also within healthcare organizations.
2.	Rea KE, Rao P, Hill E, et al (abstract only)	USA	A Systematic Review	Patients 0–21 years	Family-centered rounding (FCR) in pediatric wards	To systematically review patient and family experiences with pediatric FCR	FCR involves multidisciplinary rounds at bedside in which the patient and family are involved in creating the plan and evaluating the rounding process. ‍The providers are introduced to the model, taught and practice communication with families in to improve parents understanding of the care being provided. ‍	Family benefits of FCR included increased understanding of information and confidence in the medical team, as well as reduced parental anxiety.
3.	Khan et al (2018)	North America	Multicenter before and after intervention study	Not mentioned–pediatric inpatient units	A co-produced family centered communication programme	Medical errors (primary outcome), including harmful errors (preventable adverse events) and nonharmful errors, family experience; and communication processes (eg, family engagement on rounds).	A team of physicians, nurses, and families coproduced an intervention to standardize rounds using high reliability structured communication that emphasized health literacy, family engagement, and bidirectional communication and teamwork.	Harmful errors decreased by 38% across seven North American academic hospitals after implementation of the intervention, although overall medical errors (harmful plus non-harmful errors) did not change. In addition, aspects of family experience and communication processes improved, without negative impacts on rounds duration or teaching on rounds.
4.	Nair et al (2014)	Global	A metareview of systematic reviews	Newborn and children	*Newborn*- Training materials for CHWs prepared involving community support groups and/or women’s group *Child*- Family centered care	To identify facilitators and barriers to improving quality of care (QoC) for pregnant women, newborns and children show that training and communication improved the QoC for newborn and child health.	*For newborn-* use CHWs to in raising awareness and educating parents about newborncare. *For Child*–family centered care Provider training and communication and their effect on quality of care.	*Newborn-* Telemedicine technology focused on education and support of parents of newborns in NICU could improve parents’ satisfaction but was not effective in reducing the length of hospital stay. *For the child* lack of effective communication between providers and parents was an important process barrier that negatively affected parents’ satisfaction and their engagement in healthcare.
5.	Franck LS et al., 2019	Global	A review	Newborn 0–28 days	Family-centered care	Classifies family centered care intervention and highlight the strong level of research evidence on family centered interventions that include parent-delivered, parent focused and those that support NICU parents.	Interventions to support parents, parent-delivered interventions, and multidimensional models of NICU care that explicitly incorporate parents and partners in the care of their preterm or low birthweight infant.	1). *Parent-focused NICU interventions and parent-partnered care models;* interventions to support parents” defined as psychoeducational, communication, or environmental interventions that support parents to cope with the NICU experience and to ultimately be emotionally, cognitively, and physically able to parent their infant *2)*. *Parent- delivered interventions*, parents in NICU can safely and effectively perform if parents receive training and support from skilled healthcare providers such as basic newborn care, such as bathing, diapering, and clothing and also learn their infant’s developmental cues such as assessment of feeding readiness, signs of stress, or readiness for social interaction), and assist the NICU team in providing a developmentally supportive care environment for the infant (e.g., modulating light, sound, and touch). 3). *Interventions to support NICU parents;* designed to support parents in coping with the NICU experience so that they can ultimately be emotionally, cognitively, and physically able to parent their infant.
6.	O’Brien (2018)	Canada	Multicentre cluster-randomized controlled trial	0–59 days (NICU)	Family Integrated Care (FICare)	Infant weight gain at day 21 after enrolment. Secondary outcomes were weight gain velocity, high frequency breastfeeding (≥6 times a day) at hospital discharge, parental stress and anxiety at enrolment and day 21, NICU mortality and major neonatal morbidities, safety, and resource use (including duration of oxygen therapy and hospital stay).	Training and implementation of FICare Pillars that included: 1) Parent education and support; 2) Staff education and support; 3) Psychosocial support: 4) Environmental support: Unit policies and practices to support parent engagement including environment support for prolonged parent stay.	FICare improved infant weight gain, decreased parent stress and anxiety, and increased high-frequency exclusive breastmilk feeding at discharge, which together suggest that FICare is an important advancement in neonatal care.
7.	Purdy, Craig and Zeanah (2015)	Global	Review	0–59 newborn (NICU)	Family-Centered Care	a) emotional support, (b) parenting education, (c) medical follow-up care and (d) home visitations	A multi provider team where there is exchange of information between team members and parents is essential to identify psychosocial stress and respond to family concern about care in NICU and upon discharge to help transition to home environment.	Recognition of the emotional stressors experienced by parents and working to provide the crucial support and parenting skills is needed for bonding and caring for their infant from admission through discharge and beyond. Establishing individualized, flexible but realistic, pre- and post-discharge plans with parents is needed to start their healthy transition to home and community
8.	Sarin E and Maria A. (2019)	India	Qualitative cross-sectional survey	0–28 days, sick infants in NICU	Family-Centered Care	Acceptability of family-centered care among providers and family members of neonates to identify gaps and challenges in implementation. Specifically assessed Integration of the FCC program in the NICU routine activities, Clinical benefits of FCC, Empowerment and self-efficacy of parent, perceptions of non-compliant parents create more work.	On admission, parents are sensitized to FCC by a face-to-face session with a provider. Orientation on the concept and importance of FCC, the training process required to become a parent-attendant, and their role in care provision. Trainings include audio-visuals, role plays / skills station and informational materials. Parent attendants are trained over 4 sessions (30–45 minutes). Experienced FCC mothers were encouraged to demonstrate skills to newer mothers.	Family members and providers expressed a positive perception and acceptance of FCC based on the competencies and knowledge acquired by parents and other caregivers of essential newborn care. Family members reported being satisfied with the overall health care experience due to the transparency of care and allowing them to be by their baby’s bedside. Limitations in the infrastructure or lack of facilities at the public hospital did not seem to dilute these positive perceptions.
9.	Verma, et al (2017)	India	A Randomized Controlled Trial	Sick Newborns 0–28 days	Family Centered Care	Assessed the impact of family-centered care in delivery of care to sick newborns, on nosocomial infection rate.	Parent-attendant of intervention group were trained using an indigenously developed and pretested, culturally sensitive, simple audio-video tool that covered domains of personal hygiene, hand washing, danger signs recognition and feeding of sick neonate.	Incidence of nosocomial episodes of sepsis was not different between groups (incidence rate difference 0.74, 95% CI -4.21, 5.6, P = 0.76). Pre-discharge exclusive breastfeeding rates were significantly higher in intervention group [80.4% vs 66.7% (P = 0.007)].
10.	Uhl T, Fisher K et al (2013)	USA	mixed-method descriptive design survey	0–13 years	Patient and Family Centered Care	Describe parents’ care experiences such as communication, knowledge on child’s treatment plans during hospitalization of their children to identify strategies that could improve the provision of patient and family centered care (PFCC).	The concepts of PFCC include but are not limited to parental role negotiation, effective communication among the health care team and parents, parental decision-making processes, and continual parental presence.	Parents’ ability to engage successfully in the hospital experience was influenced by effective communication with the healthcare team. Lack of parental knowledge about their child’s treatment plans was an important gap in communication that negatively influenced parents. Parents felt that some nurses expected them to contribute to the care of their infants, whereas other nurses considered the parent a nuisance.
	Rea KE, Rao P, Hill E, et al (abstract only)	USA	A Systematic Review	Patients 0–21 years	Family-centered rounding (FCR) in pediatric wards	To systematically review patient and family experiences with pediatric FCR	FCR involves multidisciplinary rounds at bedside in which the patient and family are involved in creating the plan and evaluating the rounding process. ‍The providers are introduced to the model, taught and practice communication with families in to improve parents understanding of the care being provided. ‍	Family benefits of FCR included increased understanding of information and confidence in the medical team, as well as reduced parental anxiety.
11.	Powers S etal., 2020	United States, Europe and Israel	Scoping Review of 29 studies	0–28 Days	Parental Presence in the Neonatal Intensive Care Unit	To measure parental presence in the NICU and reported associations of presence with patient demographics, parental engagement in the NICU, and outcomes for both infants and parents.	Parental presence was defined as the proportion of days with at least one visit, but also included days per week with at least one visit or visits per day or visits per other time intervals were also used.	Main facilitators of parental presence were scheduled weekly appointments to facilitate maternal contact and familiarity with the infant, which resulted in increased independent maternal visits compared with a control group. Although another study found that sought to address cost or transportation issues did not have that much effect on parental presence.
12.	Camacho Ávila M et al., 2020	Southwest, Spain	Qualitative	0–28 days	Professional counseling and socio support to grieving parents	To describe and understand the experiences of parents in relation to professional and social support following stillbirth and neonatal death	Counseling and support according to parents’ requirements by a team of professionals	Grieving parents reported lack of continued care/support for despite sustained access to postnatal support (mental, social). Parents were particularly feeling a sense of loneliness in their experience of perinatal death—at hospital and socially (after) which compounded negative experience of loss of a child. This loss and difficulty was worse for parents who had no other children to distract them (a coping mechanism to process grief)
13.	Baughcum AE, et al., 2020	United States, Ohio	Quantitative	0–28 days	End of life care in NICU	To examine parents’ perceptions of their infant’s End of Life experience (EOL) (eg, symptom burden and suffering) and satisfaction with care in the NICU	Including parents as partners in care, communication with the health-care team, establishing relationships with staff, and bereavement support.	Mothers felt they did not fully understand the cause of death/medical aspects. They weren’t completely satisfied with health staff assistance in EOL decision-making. They were also slightly less satisfied with overall care than fathers. Both parents had low satisfication scores with their emotional needs being met.
14.	Nakphong MK et al (2020)	Kenya	Quantitative study	0–59 days	Newborn care, breastfeeding	Outcomes related to satisfaction with care and care utilization, 2) Continuation of post-discharge newborn care practices such as breastfeeding.	Not provided	17.6% of women reported being separated from their newborns at the facility after delivery, of whom 71.9% were separated over 10 minutes. 44.9% felt separation was unnecessary and 8.4% reported not knowing the reason for separation. 59.9% reported consent was not obtained for procedures on their newborn. Women separated from their newborn (>10 minutes) were 44% less likely to be exclusively breastfeeding at 2–4 weeks (aOR = 0.56, 95%CI: 0.40, 0.76).
**Family/Parental engagement**								
1.	Ballantyne M et al., 2017	Global	A scoping review	0–12 months Preterm or ill infants	(i) enhanced parent engagement; (ii) information-sharing, communication and shared decision-making; and (iii) capacity-building to parent and navigate the future.	Provider- parent response to infant’s transition/change within and between hospitals and across levels of neonatal intensive care unit, intermediate and community hospital care.	Provider parent engagement, communication, and information-sharing and capacity building for parent to help prepare for future with health care providers	Results also shows that parents’ stress resulted from not being informed or involved in the transition decision, inadequate communication and perceived differences in cultures of care across healthcare settings. Parenting at a distance and clack of emotion were sources of stress.
2.	Skene C et al., 2012	United Kingdom	Focused ethnography	0–28 days	Parent caregiving interaction with their infants	Explore how parents interact with their infants and with nurses regarding the provision of comfort care in a Neonatal Intensive Care Unit (NICU).	Parents were observed during a caregiving interaction with their infants and then interviewed on up to four occasions.	Parental involvement in comfort care can aid the process of learning to parent in NICU and may also facilitate the transfer of responsibility from nurse to parent and parent/infant attachment.
3.	Carman, K.L., et al., 2013	USA	A review	Not specified	Describe a framework of patent and family engagement that occurs across the health system.	Examines the levels at which patient engagement can occur throughout the health care system, in direct care, organizational design and governance, and policy making.	Training providers to support patient engagement and partnering with patients at the organizational level to plan, deliver, and evaluate care also influence the family engagement.	Patient engagement depends on how much information flows between patient and provider, how active a role the patient has in care decisions, and how involved the patient in health organization decisions and in policy making. At the continuum’s lower end, patients are involved but have limited power or decision-making authority. Providers, organizations, and systems define their own agendas and then seek patients’ input.
4.	Celenza et al., 2017	USA	A review	0–59 days	Family Involvement in Quality Improvement	Different approaches and strategies to engage families as partners in NICU QI efforts.	Family or their representatives are engaged in every aspect of the hospital system to enable teams and partnership mechanisms. Families involvement in codesigning and co-leading quality improvement projects and have representation on committees and may leverage advisory councils for feedback and assistance.	Involving families in NICU as stakeholders in quality improvement enhances partnerships with families and seeking to improve this key relationship to nurture a culture that ensures the best possible neonatal outcomes.
5.	Eden & Callister, 2010	UK, USA, Scotland, Sweden, Switzerland and Canada	An integrative literature review	Newborn	Parent Involvement	To evaluate parental involvement in end-of-life care and decision making for their infant in the NICU	Palliative care programs provide support for parents and facilitate their decision making. Parents education about how to communicate with health-care providers. Educating nurses on how to provide end-of-life care in order to improve support for parents during this difficult time.	Findings revealed that establishing good relationships and clear communication between providers and parents builds trust and eases stress placed on parents making decisions about the care of their infant.
6.	Melo et al., 2014	Portugal	An exploratory qualitative study	Pediatrics 6 weeks -5 years	Parent Involvement	To assess the understanding of parents and health care professionals on involvement of parents in the care provided to hospitalized children.	Providers and parents see this as daily interactions between them in the process of providing/receiving care. Communication between parents and health care professionals and facility infrastructure. Involvement of parent in responsibility and right to perform of care activities while at the hospital and continuity of care after discharge.	The involvement of parents in the care provided to their children has many meanings for parents, nurses and doctors. To parents, communication is very important in family involvement, while providers thought that orientation and focused health education and training of parents to provide care to children is key.
7.	Richter et al (2012)	South Africa	Intervention (pilot) study	Not mentioned–pediatric ward	Improving nursing care of young children in a HIV/AIDS area through engaging parents	Addressed caregiver expectations about admission and treatment, responsive feeding, coping with infant pain and distress, assistance with medical procedures, and preparation for discharge and home care.	The intervention package included five, short educational videos created to demonstrate to nursing staff and caregivers’ solutions to difficulties in caring for hospitalized children affected by HIV/AIDS from extensive naturalistic video recordings made of daily care in the ward.	No changes were found between before and after intervention on assessments of caregiver wellbeing. However, mothers in the postintervention phase rated nurses as more supportive; mother-child interaction during feeding was more relaxed and engaged, and babies were less socially withdrawn. While the intervention proved useful in improving certain outcomes for children and their caregivers, it did not address challenging hospital and ward administration, or support needed by caregivers at home following discharge.
8.	So. S et al (2014)	Canada	Evaluation study	1–15 months	Beanstalk Program to support parental involvement and developmental needs of children in hospital	Extent to which certain behaviors of health-care providers occur and is widely applicable for measuring parent’s perceptions of family-centered caregiving, regardless of the child’s diagnosis or specific are such as what enabled partnerships, information provided, if care was coordinated and comprehensive.	Parents and interdisciplinary team members (nurses, physicians, physiotherapists, occupational therapists, social workers and child life therapists) were provided with ongoing education on normal development and age-appropriate interactions/play strategies. Parents were then provided parental education about normal development and impact of illness/hospitalization specific to their child. To encourage the collaboration of interdisciplinary team members in met to developmental needs of the children.	Results were overwhelmingly positive, with parents perceiving high levels of supportive care during their child’s prolonged hospitalization. However, parent reported receiving in adequate information and wanted more about services available at the hospital, the BP, the child’s chronic illness and its resultant impact on development
9.	Tokhi et al (2018)	Netherlands	A review	Not Mentioned	Interventions to engage men during pregnancy, childbirth and infancy on mortality and morbidity	Male partner support for women including breastfeeding; couple communication and joint decision-making and effects on women’s autonomy.	Length of interventions ranged from five months to 12 years and delivered through diverse mechanisms including community outreach and education, home visits, facility-based counselling, workplace education programs and mass media social mobilization campaigns.	The impact of interventions that involved men on breastfeeding was less clear but there was improved care for those that engaged men in joint decision making on facility birth, postpartum care, birth and complications preparedness and maternal nutrition
10.	Camacho Ávila M, et al., 2020	Southwest, Spain	Qualitative	0–28 days	Professional counseling and socio support	To describe and understand the experiences of parents in relation to professional and social support following stillbirth and neonatal death.	Case study conducted on parents immediate services after the loss of newborn or still birth and continuing care at home	Grieving parents reported lack of continued care/support for despite sustained access to postnatal support (mental, social). Parents were particularly feeling a sense of loneliness in their experience of perinatal death—at hospital and socially (after) which compounded negative experience of loss of a child. This loss and difficulty was worse for parents who had no other children to distract them (a coping mechanism to process grief)
	Baughcum AE, et al., 2020	United States, Ohio	Quantitative	0–28 days	End of life care in NICU	To examine parents’ perceptions of their infant’s End of Life experience (EOL) (eg, symptom burden and suffering) and satisfaction with care in the NICU	Including parents as partners in care, communication with the health-care team, establishing relationships with staff, and bereavement support.	Mothers felt they did not fully understand the cause of death/medical aspects. They weren’t completely satisfied with health staff assistance in EOL decision-making. They were also slightly less satisfied with overall care than fathers. Both parents had low satisfication scores with their emotional needs being met.
11.	Maatman S et al (2020)	Sweden, Norway and Netherlands	Qualitative study	0–59 days	Family Centered Care in NICU	Factors influencing implementation of Family-Centered Care NICU’s among three different northern European countries	All included hospitals implemented FCC and subsequently rebuild their wards between 2010 and 2012; including rooming-in or sleeping facilities for parents near their infants.	Four aspects were identified, when analyzing the data, namely: Behavioral change in staff, Family needs, environment, and Communication. Most important is that almost all healthcare professionals described that the mind-set of the professional influences the implementation of FCC.
12.	Klug et al (2020)	USA	Quantitative study	0–12 months	Promoting parent partnership in developmentally supportive care using a visual tool called the Care Partnership Pyramid	i)Parent Partnership from nursing notes the Outcome measures were 1)parents participating in rounds 2) asking appropriate questions 3) providing environment comfort 4)providing appropriate developmentally supportive stimulation 5) changing diapers 6) assisting with daily care routing and 7) holding the infant. ii)An electronic survey was distributed to staff in the CICU with 2 primary question "how useful they think the tool is for care of the patient" and "how useful they think the tool is for the family?" and staff perceptions of a visual tool	The Care Partnership Pyramid was printed, laminated and hung on the welcome board on the bedside for use by the family and care team. Families were oriented to the tool upon admission. Three "Plan-Do-Study-Act" (PDSA) cycles tested the impact of the intervention on parent partnership in care	After 3 cycles of the "Plan-Do-Study-Act"—parents were more often observed participating in rounds, asking appropriate questions, providing emotional comfort, assisting with daily care routines, and changing diapers. Staff perceived that the tool was generally useful for the patient and the family but was sometimes overlooked or not used. Use of a bedside visual tool may lead to increased parent partnership in care for infants after cardiac surgery
13	Naef R et al (2020)	Switzerland	Mixed methods	0–59 days	Family systems care (family centered care)	1)What is the impact of family systems care implementation on practitioners’ attitudes towards families, and their practice skills in working with families; (2) How do practitioners experience implementation of this new knowledge into practice?	The inter-professional approach family systems care (FCC model) involved family meetings throughout an infant’s care process and follow up. The intervention, implemented over 8 months involved an educational workshop (3 sessions over several weeks).	A statistically significant increase in practice skills and reciprocity, but not in attitudes was found mid- and post-implementation. Practitioners reported new ways of working with families, which included enhanced awareness of the extended family, intentional relationship-building, augmented family involvement, and systemic interventions, such as therapeutic listening. They experienced implementation as a wheel that moved forward or stood still, depending on the challenges faced and the predominance of enabling versus limiting organizational factors.
	Nakphong MK et al (2020)	Kenya	Quantitative study	0–59 days	Newborn care, breastfeeding	Outcomes related to satisfaction with care and care utilization, 2) Continuation of post-discharge newborn care practices such as breastfeeding.	Not provided	17.6% of women reported being separated from their newborns at the facility after delivery, of whom 71.9% were separated over 10 minutes. 44.9% felt separation was unnecessary and 8.4% reported not knowing the reason for separation. 59.9% reported consent was not obtained for procedures on their newborn. Women separated from their newborn (>10 minutes) were 44% less likely to be exclusively breastfeeding at 2–4 weeks (aOR = 0.56, 95%CI: 0.40, 0.76).
**Provider communication and counselling**								
	Ballantyne M et al., 2017	Global	A scoping review	0–12 months Preterm or ill infants	(i) enhanced parent engagement; (ii) information-sharing, communication and shared decision-making; and (iii) capacity-building to parent and navigate the future.	Provider- parent response to infant’s transition/change within and between hospitals and across levels of neonatal intensive care unit, intermediate and community hospital care.	Provider parent engagement, communication, and information-sharing and capacity building for parent to help prepare for future with health care providers	Results also shows that parents’ stress resulted from not being informed or involved in the transition decision, inadequate communication and perceived differences in cultures of care across healthcare settings. Parenting at a distance and clack of emotion were sources of stress.
1.	Ali M et al 2018	Middle East—Iraq	Cross sectional quantitative survey	Children under 5	Implementation of IMCI components	Impact of training on practical IMCI skills that include ability to assess, classify and treat illness in children.	Provider training, in IMCI influence on knowledge and practice on IMCI and practice/ adherence to ensure that children receive evidence-based care according to WHO guidelines	Training has a positive influence on the implementation of IMCI interventions. IMCI-trained caregivers were more likely to correctly classify illnesses than non-trained caregivers. Supportive supervision and periodic training courses to IMNCI-trained caregivers improved providers practice. Recording and mother instructions skills among caregivers and giving required more attention. There was lack of active referral systems and feedback on nutrition at level of PHC centers, general hospitals and directors.
2.	Chan G. et al., 2017	Global	Review	0–28 days	Kangaroo mother care (KMC)	Evaluates barriers and facilitators to Kangaroo baby care	Review reported use of technology health workers KMC workshops and use of cell phone messages encouraging KMC implementation.	Lack of manpower, interactions between heath workers, training, communication, and support were barriers to the adoption of KMC. Acceptance of KMC by facility leadership, increase resource allocation to KMC within the facility and prolong visitation hours facilitated KMC implementation
3.	Lucas et al., 2017	Global	Evaluation/review of evidence	0–5 years	Care for Child Development	Care for Child Development (CCD) that focus on sensitivity to children’s movements, sounds and gestures and interpreting and responding appropriately to them. Responsive caregiving in protecting children against injury, recognizing and responding to illness, enriching learning and building trust and social relationships.	Counsellors ask caregivers how they play and communicate with their children, how they get their children to smile and how they think their children are learning. The counsellor observes how the caregiver responds, comforts, shows love and guides the child’s exploration. The counsellor uses the information to praise the caregiver, build the caregiver’s confidence, increase child-directed language and identify enjoyable activities that the caregiver and child can do together at home.	Evidence-based intervention CCD is effective in improving responsive caregiving practices and child health and development outcomes, psychological wellbeing of the child and to reduce maternal depression. It is feasible to implement at relatively low cost
4.	Bucher, H.U., et al., 2018	Switzerland	A survey among neonatologists and neonatal nurses	0–28 days	End of life decision making	To analyze practices, difficulties and parental involvement in end-of-life decisions for extremely preterm infants.	An online survey with 50 questions on end-of-life decision-making.	Difficulties with end-of-life decision-making were reported more frequently by nurses than physicians. They included insufficient time for decision-making, legal constraints and lack of consistent unit policies. Nurses were more reluctant to give parents full authority to decide on the course of action for their near-death infant.
5.	Goggins et al (2016)	Kenya	Randomized Clinical Trials (RCT)	6–12 weeks	HIV Infant Tracking System web-based intervention	A patient tracking system with text reminders for mothers—to improve communication and accountability of all stakeholders once infants enrolled in Early Infant Diagnosis programme (EID) services.	Education to mothers/parents on HIV early infants’ diagnosis during antenatal visits, postnatal follow up at 6 and 12 weeks for babies born to mothers living with HIV.	Findings highlight the importance of ensuring that health care providers in actively and repeatedly inform HIV mothers of the availability of EID services, reduce stigma by frequently communicating judgment free support, and assisting mothers in early planning for accessing EID services.
6.	Gondwe et al 2017	Malawi	Qualitative Survey	Sick newborn 0–28 and infants 29 to 180 days	An innovative, low-cost bubble continuous positive airway pressure (bCPAP) device	Information on care plans including use of bCPAP; Perceptions/feelings on bCPAP; Psychological support during care.	Information was provided to parents on what bubble bCPAP devices are and why their babies were on them. This aimed at reducing anxiety and fear of the care being received.	Information provided was reported to be inadequate, but caregivers received psychological support from healthcare workers, family members, and friends. caregivers perceived psychological support from family members as vital to their psychosocial well-being during bCPAP
7.	Flenady V. et al 2014	USA	A review of evidence	Newborn	Counselling and other therapeutic interventions such as respect for the individuality and diversity of parents’ grief around parent-centered printed materials.	The grief of mothers, fathers and families, social stigma and negative attitudes associated to babies’ deaths, underreporting of babies’ deaths in low- and middle-income countries, a failure to recognise the value of these lost lives (newborns)	Providing objective information about newborn death in a calm, supportive manner where critical information should be repeated and reinforced. Creating memories such as holding, bathing and dressing the baby, talking to the baby and using the baby’s name, engaging in religious or naming ceremonies and capturing interactions in photographs and movies would support mother and families deal with grief after baby’s death.	Provider training to ensure that they are equipped to provide appropriate care following a perinatal death to help parents cope with stress in the critical period. There is need for improved reporting newborn deaths.
	Khan et al (2018)	North America	Multicenter before and after intervention study	Not mentioned–pediatric inpatient units	A co-produced family centered communication programme	Medical errors (primary outcome), including harmful errors (preventable adverse events) and nonharmful errors, family experience; and communication processes (eg, family engagement on rounds).	A team of physicians, nurses, and families coproduced an intervention to standardize rounds using high reliability structured communication that emphasized health literacy, family engagement, and bidirectional communication and teamwork.	Harmful errors decreased by 38% across seven North American academic hospitals after implementation of the intervention, although overall medical errors (harmful plus non-harmful errors) did not change. In addition, aspects of family experience and communication processes improved, without negative impacts on rounds duration or teaching on rounds.
8.	Kavle et al., 2019	Nampula, Mozambique	Implementation science study	0–6 months	Use of job aids in counseling on Exclusive breast feeding (EBF)	EBF challenges, from the perspectives of health providers and mothers; quality of health provider counseling to address EBF challenges; and gain an understanding of the usefulness of job aids to improve counseling within routine health contact points	Health providers were trained to use three job aids (i.e., facility, community or maternity contacts) to identify and address EBF problems during routine health services	Provider and mothers as well as integration of job aids, with clear lactation management guidance, into maternal and child health training curricula and supportive supervision is critical to building providers’ skillsets and competencies to provide quality lactation counseling and support.
	Nair et al (2014)	Global	A metareview of systematic reviews	Newborn and children	Newborn- Training materials for CHWs prepared involving community support groups and/or women’s group Child- Family centered care	To identify facilitators and barriers to improving quality of care (QoC) for pregnant women, newborns and children show that training and communication improved the QoC for newborn and child health.	For newborn- use CHWs to in raising awareness and educating parents about newborn care. For Child–family centered care Provider training and communication and their effect on quality of care.	Newborn- Telemedicine technology focused on education and support of parents of newborns in NICU could improve parents’ satisfaction but was not effective in reducing the length of hospital stay. For the child lack of effective communication between providers and parents was an important process barrier that negatively affected parents’ satisfaction and their engagement in healthcare.
9.	Psaila K etal., 2014	Australia	An exploratory descriptive survey	0–28 days (postnatal mother)	Transition of Care for new mothers to provide continuity of services from facility to home care after discharge.	Transfer of information, time of CFH nurse first contact with new clients, frequency, completeness of documentation and when, the information is provided, and effectiveness of information from maternity services, home visiting services and frequency of contacts and routine psychosocial assessment upon discharge	Midwives provide childcare follow up information at discharge and conducted postnatal home visits to provide support on care and transmitted reports on their activity in hard copy to the facility.	Lack of communication between domiciliary midwifery care and child and family health (CFH) centres. CFH nurses often unable to take phone call from domiciliary nurses due to workload. It was also more difficult to communicate with families with identified social and emotional health concerns
10.	Richardson B, Falconer A et al 2020	Canada, UK, Brazil	A review	0–28 Days	Parent education on pain management	To explore and map the current evidence of parent-targeted educational interventions about infant pain, delivered throughout the perinatal period	Parent-Targeted Education Regarding Infant Pain Management Delivered During the Perinatal Period"	interventions reviewed contained information about parent-led pain management strategies for infants in the neonatal intensive care unit (n = 4), full term (n = 4), or both (n = 1). Despite being an area of high concern for parents of newborns, few studies addressed parent-targeted education regarding infant pain.
11.	Treyvoud K, et al. 2019	Global	A review	0–28 Days	Parents support in NICU	Reviews the effectiveness of interventions for infants and children born preterm	Reviews found that the interventions included parents’ peer-to-peer support between parents such as face-to-face meetings, phone, group, and online communication forums for distressed parents and training NICU providers on mental health	A multilayered approach to supporting parents of infants born preterm in the NICU is recommended, with evidence specifically for including layers of individual psychological and psychosocial support, peer-to-peer support, and family centered care.
	World Health Organisation (2020)	Global	None	0–28 days	Baby Friendly Hospital Initiative for Small, Sick and Preterm Newborns	None–not a study	Implementation of ten steps to successful breastfeeding in health facilities	Some of the key clinical practices included: Skin-to-skin care, kangaroo mother care, family-centered care, providing milk and breastfeeding empowers mothers to become the primary caregivers of their infant. Also mentions rooming in where mothers and babies stay together 24 hours a day. Responsive feeding where mothers are taught and can identify feeding cues

### Experience of care for newborns and sick young children (0–24 months) and their caregivers

Negative experiences of care manifest in various forms and were described in eight studies (Table 1). Mistreatment of newborns is described by Sacks [15] who expands on a typology for mistreatment during labour and delivery outlined by Bohren et al [10]. The resulting mistreatment categories include: physical abuse; verbal abuse; stigma and discrimination; failure to meet professional standards; poor rapport between providers and patients; and health system conditions and constraints; legal accountability and poor bereavement and posthumous care [15]. We were unable to find any papers with a specific study purpose of describing mistreatment of young children (60 days– 24 months) who are hospitalized or receiving care at health facilities.

Negative provider attitudes manifest as unfriendly verbal expressions or lack of attendance to parents when they complain about care. This may lead to distrust in the health system and influence women’s future care-seeking decisions for future childbirth at facilities, postnatal care, and accessing essential child health services [21]. Health systems constraints such as limited space for mothers’ accommodation, noisy, brightly lit, and intimidating units or wards, without much privacy and strict visitation protocols result in long periods of separation between mothers and infants, which is shown to be common in the neonatal intensive care unit (NICU). This might be stressful and traumatic for the mother and newborn, as it hinders bonding and leads to long-term consequences for the mother-baby relationship and may also impede breastfeeding [22–24]. Other experiences described include unjustified lengthy admissions, over diagnosis of risk conditions (for example, predicting urinary tract infection in many infants while only a few actually have it), inappropriate use of drugs often with unnecessary intravenous or intramuscular treatments for both mothers and newborns in management of life-threatening complications, and disregard of women’s right to information, privacy or confidentiality [23].

Negative experiences also occur as a result of poor provider communication with parents on caring for their hospitalized young children (n = 9). Studies show that women, were often poorly informed about clinical practices and procedures, lack autonomy to make treatment choices for their newborns; in cases of small and sick newborns, this can lead to emotional crises including maternal post-traumatic stress, anxiety and depression [16, 23, 25–27]. Other studies show that limited communication between midwives and parents and families may be due to a provider’s heavy workload [28, 29]. One study suggests that parents who experienced a stillbirth felt ignored by providers’ and perceived providers’ inattentiveness as “normal but troubling” while providers describe this as a “coping strategy” to avoid difficult conversations with parents [30]. In another study, providers had difficulty integrating parents of babies with congenital anomalies in nursing care activities [31]. Elsewhere both provider and parents’ perspectives were identified. One study showed parents perceived providers as being rude or withholding information or not listening to mothers; non-consented care; speaking loudly about baby’s condition without consideration for privacy and confidentiality. On the other hand providers’ described mothers not following instructions (not washing hands prior to entering neonatal unit); giving incorrect or misleading information "lying about the condition" to have their baby discharged sooner [32].

Positive experiences of care, across (n = 12) studies, were reported where parents were able to express their opinions’ on care given to their newborns and when there was good communication between providers and parents [22, 26, 33, 34]. Positive interactions were evident when parents are taught basic nursing skills such as feeding, cleaning, clothing and monitoring their newborn,.[22, 27, 35]; and where care plans and clinical decisions (particularly low risk ones) are made jointly by parents and providers, and consider the context of the family and community [35]. Positive experience was also reported if parents were allowed to provide comfort to their newborn and assist with daily routine care in an encouraging and enabling environment [36, 37]. Processes that support mothers and fathers to learn how to care for their premature babies while still in hospital in readiness for caring for their babies at home was described as a positive experience of care. This helped parents overcome their fears and insecurities, strengthen mother-infant bonds, and made them feel empowered to participate actively, in the care of their babies and increased confidence of caregiving at discharge [32, 34]. Positive experiences of parents, including providers responding to their needs and rights, was also instrumental in improving a child’s ability to thrive. For hospitals with limited resources, having parents perform some tasks such as tube feeding helped to address staff shortages in nurseries, and functioned as a short-term economic savings to the facilities [22]. Positive experiences were also reported where parents with sick newborns in NICU were helped to meet their emotional support needs and information on care through parents support groups [38]. There were no specific studies examining experience of care among young children beyond 59 days.

### Drivers of negative experience of care

Twelve studies describe the various factors influencing or “driving” negative experiences of care within health systems and among communities, and from individual providers.

Under-resourced health systems, and specifically at facility level, contribute to drivers of patient mistreatment. Often due to weak infrastructure and limited equipment, including lack of such basic elements such as running water, crowded units with limited bed capacity (where newborns are often sharing incubators or cots), noisiness, lack of privacy, lack of life saving commodities and facility policies, poor adherence to policies and guidelines and poorly trained staff are especially acute factors.

In a multi-country study, readiness for skin-to-skin implementation in newborn units was extremely rare [39], and failures in meeting standards of care and professional conduct, examples, patient neglect, poor staff accountability, non-consented care, and poor bereavement care all contribute to mistreatment of newborns [15, 30]. A study in Southwest Spain and another in United States, found that lack of continued care/support (mental, social) for grieving parents who had experienced a stillbirth or neonatal death led to feelings loneliness, and a lack of understanding and satisfaction with end-of-life care provided [40, 41]. While provider attitudes and norms appear to influence behaviors that can be described as mistreatment, or produce negative experiences for parents or young children, no studies focused on examining these factors. Poor experiences of patient care may also result from socio-cultural factors and community norms for the care of newborns and young children. In some LMICs, both provider and familial neglect of newborns and young infants at a facility may derive from societal non-recognition of newborns as full “human beings” [42] or prioritizing a mother’s needs over newborns and lack of access to health care [43]. Practices such as delayed initiation of breastfeeding due to perceived lack of milk, or because a baby “needs to sleep” after delivery, or does not show outward signs of hunger, are examples of socio-cultural factors [43–45]. A multi country review found that health workers who lack knowledge and training on the importance of early and timely breast expression do not support and educate the mother on it [20]. A study in Gujarat, India found that skin-to-skin care was not practiced in most cases regardless of place of delivery, and immediate breastfeeding was not practiced in almost 63 percent of all deliveries, because most mothers believed colostrum digestion is difficult for newborns, or because the infant is kept from the mother after birth [46]. Data from Ghana and Ethiopia show that cultural norms such as early bathing can be prevalent even in facilities [20]. Another study in Ethiopia reported practices of bathing the newborn during the first 24 hours of life (75%), application of butter and other substances to the cord (20%) and discarding colostrum milk (44.5%)—all three contrary to WHO recommendations [47].

### Interventions to improve positive experience and emotional needs of parents with sick young children

This scoping review found 40 studies of interventions that may improve experiences of care, reduce mistreatment, or improve provider and parent communication and partnership whereby parents are central to caregiving. Of these, 14 were review articles, three were randomized control trials three pilot interventions, one was an implementation study, and two were evaluations.

In this review we refer to the WHO’s Nurturing Care Framework [6], as it provides global guidelines on important strategies for access, provision, and more explicitly, experience of care that place parents, providers, managers and policymakers as central to quality services.

The interventions discussed in this review are not mutually exclusive but focus on encouraging positive interactions between providers and parents. Some interventions emphasize the use, adaptation, or development of policy guidance, care protocols and job aids, parental or provider trainings, and use of technology to support an implementation. Nurturing care emerged as an approach that enhance physical, emotional and cognitive development. To effectively implement nurturing care three main interventions that can enhance nurturing care emerge from this review that may improve the experiences of care for newborns and young children: nurturing care, family-centered care, strategies to enhance parental/family engagement which all underscores provider communication and counseling skills.

#### a. Nurturing care approaches

Three studies; all of which are global reviews and one WHO framework describe approaches to ensure nurturing care. Nurturing care is defined by Britto et al. as *“a stable environment that is sensitive to children’s health and nutritional needs*, *with protection from threats*, *opportunities for early learning*, *and interactions that are responsive*, *emotionally supportive*, *and developmentally stimulating”* [48]. Interventions encompassing nurturing care have been predominantly in high income countries, throughout the life continuum, starting in pregnancy with education and counseling, continuing through childbirth, the newborn period, infancy, and early childhood. Elements of a nurturing care approach might ensure, for example, skin-to-skin care for small and sick newborns, optimal nutrition (i.e. breastmilk feeding and exclusive breastfeeding, infant and young child feeding), and protection and promotion of sleep. Other elements of nurturing care include reducing stress and pain, supportive positioning, sensory environment, stimulation, and interaction.

In the long term, nurturing care promotes a child’s physical and cognitive development, reduces morbidities and mortalities, and prevents disability injuries and deaths [6, 16, 20, 48]. Nurturing care provides neuroprotection in NICU as well as enhances parental ability to read and understand the behavior of the immature infant, and to support the responsive parent and infant bond. Despite its small and sick newborn focus, nurturing care elements such as adequate rest, reduced sensitivity and motor activity (adequate play, quite/calm environment) are appropriate for older infants and young children who are unable to articulate their needs (up to 24 months of age).

This review shows that nurturing care during management of pre-term births and those born with complications is associated with outcomes such as early bonding, facilitated by skin-to-skin contact, and early initiation of breastfeeding, facilitated by a companion to support the new mother [6, 20]. Other benefits include reducing pain immediately after a procedure through touch or massage, non-nutritive sucking, and a calm environment, with reduced noise and light levels in the NICU are associated with reducing stress. Additionally, skin to skin contact 30 minutes before a painful procedure and sweet tasting solutions, swaddling/facilitated tucking, or a combination of facilitated tucking with nonnutritive sucking are effective in reducing immediate pain and distress in inpatient preterm newborns [20]. Nurturing care is also associated with improved infant medical outcomes, staff and family satisfaction, and decreased NICU lengths of stay and hospital costs [16]. Implementation of nurturing care may include establishing teams in newborn units, training through e-Learning, didactic education, interactive workshops, physician sessions, and in-unit consultation to all individuals who care for premature infants in a NICU [6, 16, 20]. Elements of nurturing care and protection can be combined with other existing child health interventions that offer parenting support and skills development [48].

#### b. Family centered care

Family-centered care (FCC), described in 14 studies, places parents, families and caregivers at the center of newborn and young childcare. Family centered care is an approach to care delivery that promotes a mutually beneficial partnership among parents, families and healthcare providers to support health-care planning, delivery and evaluation. The principles of family-centered care include: dignity and respect; information sharing; participation; and collaboration [49]. This model resulted from increased recognition of the importance of meeting young children’s psycho-social and developmental needs, which include the influence of their families on their wellbeing [25]. FCC enhances nurturing care and is mainly described as working with parents of small and sick newborns, premature infants, and young children requiring long term hospitalization, or those with chronic illness, but is also applicable to older infants and young children.

FCC involves time-sensitive dual communication between parents and multi-disciplinary team members who coordinate care transition through emotional, educational, medical, and home visit support for families. FCC is also an approach that promotes mutual benefit among parents, their families and providers through dignity and respect; information sharing; participation; and collaboration [50]. Implementation of FCC requires some parental literacy, and willingness of parents and providers to work together and spend time in NICU or pediatric units. The model presumes providers are able to coach and educate families on specific tasks, including taking temperature, weighing, nasal gastric tube feeding, Kangaroo Mother Care (KMC), breastfeeding, hygiene, and interaction with their children including neurodevelopmental care, responsive care, among others [51].

FCC promotes a healing and nurturing environment that fosters care and protection for better linguistic, cognitive, motor, social, and emotional development, and improved psycho-social outcomes of young children under five years of age. FCC depends on providers’ knowledge and abilities to apply FCC core concepts during their interactions with parents. A randomised controlled trial evaluating FCC of sick newborns admitted in a NICU showed that babies of mothers or families trained and involved in FCC showed lower rates of nosocomial infections than a control group (not trained in FCC) though not significant [52]. FCC improved infant weight gain, decreased parent stress and anxiety, and increased high frequency exclusive breastfeeding upon discharge from a NICU [53].

Studies show that family members report satisfaction with their health care experiences involving FCC due to the transparency of care, allowing parents to be at the infant’s bedside [54], and providers demonstrating respect by listening to and honoring family perspectives and choices [20, 55]. FCC offers effective psycho-social support to parents of children in the NICU by identifying parents’ concerns and stressors for their infants’ care and developing individualized pre- and post-discharge plans [50]. FCC increases parental presence, improves physical and mental development growth, and prevents abuse and neglect of infants [56]. Implementing FCC necessitates training both for providers and parents [20, 48, 57], and often FCC interventions are focused on clinical aspects such as parents counseling on care and their participation in clinical rounds with less attention on provider introspection of their own attitudes or biases towards parents. An inter-professional approach to FCC was found to be useful in developing practical skills such as ways of working with and engaging families, increased reciprocity in therapeutic relationship, but it did not appear to change provider attitudes toward families [40].

#### c. Parental/family engagement

Eight (n = 13) studies describe parental engagement as a promising strategy to enhance effective parent and provider communication for meaningful participation in their hospitalized child’s care. WHO’s Framework for Improving the Quality of Pediatric Care recommends effective provider and parent communication and meaningful parental participation [5]. Parent engagement refers to the presence and participation of a parent (maternal or paternal) during infant care such as in comforting their infants and performing some specific care aspects [58]. While family engagement also known as family integrated care (with trained parents as mentors to other parents and family members) is a set of activities that grounds childcare in a supportive relationships and environment at the facility and home [55]. Carman et al. describes a family engagement framework of three levels, namely direct care, organizational design and governance, and policy. Each level along the continuum of care mandates consultation, involvement, partnership, and sharing of leadership [59].

Models, which educate health providers on how to communicate and provide end-of-life care, are particularly beneficial in dealing with bereavement or events after the death of a child [6, 16, 55, 60]. Parent engagement models include unplanned and informal delegation of care to parents, as well as sufficient parental training and parents’ willingness to participate in the care of their sick children [61–63]. In very sick newborns and young children it helps support parents during a difficult period. In addition to empathetic care, providers or social-workers encouraged ritual and grieving processes such as sufficient time to ‘say good-bye’ to an infant, keeping a memento, or offering space for parental expression of and communication around grief in the hospital setting were found to be helpful [40, 41]. Studies on bereavement support suggest a need for providers to be flexible in their approach to meet parental variability and cultural nuances around losing a child.

Family and parental engagement has also been associated with greater acceptability of exclusive breastfeeding, immediate breastfeeding after birth, and skin-to-skin care and infant warmth. Parental engagement also enhances families’ involvement in decisions, when parents are well-informed of what is expected of them for the care of their children in newborn units [64]. The element of peer-to-peer support provides physical caregiver wellness such as sleeping and sitting spaces and a room for procedures co-led by parents [57, 65, 66].). Parental engagement in high-income settings has been facilitated through active use of visual tools and mapping activities during a young child’s hospital stay [36, 67]. Provider behaviour change and their mindset is critical in seeing parents as primary caregivers and influences how FCC is practiced [68] though adaptions to LMICs and more socio-cultural contexts is needed.

One challenge to parental engagement strategies is the power imbalances between parents, families and their clinicians, with providers’ values and preferences potentially hindering consideration of families in decisions during care [69]. A study in Kenya found that 8% of women reported being separated from their newborns at the facility after delivery, of whom 80% were separated for more than 10 minutes. Just under half (45%) felt separation was unnecessary while 60% reported consent was not obtained prior to procedures conducted on their newborn [70]. This limited parent engagement in breastfeeding and satisfaction [70] The values and attitudes of providers within parental engagement strategies are not sufficiently considered in the interventions described.

#### d. Provider communication and counselling

Central to nurturing care, family centered care and family/ parental engagement as well as other child health interventions is the quality of provider communication and counselling. Several studies (n = 13) focused on strategies to improve communication between providers and parents. Effective communication between providers and parents is integral to improve health care, reduce medical errors, and better health care seeking. Parents play unique and integral roles in judging their child’s symptoms, and they need to be given information on care during their child’s hospital stay to ensure adequate care and follow up at home [6].

A global systematic review found that parental capacity, confidence, and communication with providers is key for joint decisions for pre-term and acutely ill infants as they transition within or between health care settings [71]. Few studies, that do so examine shared decision making in a pediatric care setting especially for newborns and acute situations, e.g., treatment decisions for high-risk newborns and end of life [72] and targeted parent education and involvement on infants pain management [73]. In Malawi, parents with inadequate and inconsistent information of when their newborns were on bubble continuous positive airway pressure (CPAP) devices were more anxious and fearful [74]. For older children, a study in Norway, found that joint decision-making increases parents’ sense of security and control of their child’s health care [75]. A study of engaging men during pregnancy, childbirth, and infancy found that enhanced communication increased couples’ joint decisions-making, leading to responsive care seeking behavior and home care practices [76]. A study in Kenya found that, parents informed early of their infant’s HIV diagnosis resulted in a feeling of less stigmatization from providers [77].

A pediatric unit study found that although a structured communication intervention for families and providers did not change, the overall rate of medical errors (per 1,000 patient days) such as administering penicillin to a patient with a known penicillin allergy, *harmful* errors (preventable adverse events) such as delay in treatment or skin breakdown from oxygen tubing decreased significantly, by 38 percent post-intervention [78]. A review found that psychological and psychosocial support can improve communication with parents. This includes peer-to-peer support between parents such as face-to-face meetings, phone, group, and online communication forums for distressed parents and training NICU providers on mental health [79].

A study in Brazil and a systematic review conducted in the United States found that involving parents during medical ward rounds, in discussions with clinicians about their sick children’s care, was instrumental in improving communication and understanding of information—and confidence in the medical team [35, 80]. Parents who reported better communication were more likely to return to the facility, while poor provider communication was associated with lower satisfaction of services received [81]. In an intervention where providers observed and provided feedback to parents, enhanced care practices of parents led to their young children’s survival, healthy growth, and physical, intellectual, language and emotional development [82].

Most interventions on communication counselling centered around information-sharing and provider development of parental capacities for specific skills through routine clinical counseling, observation and recommendations for care practices, and use of technology to promote follow up. A meta review observed that health workers should receive training in communication skills during their pre- and in-service health education. Implementation of most parent-targeted interventions emphasize regular interpersonal communication between parents and providers, with respect, confidentiality, comfort and support during care, engaging clients in care decisions, with continuity of care and audit and client response mechanisms [83].

A study in the Middle East on implementation of the Integrated Management of Newborn and Child Initiative (IMNCI) for children under five years of age found that, although clinician competency improved for assessing, classifying, and treating illnesses according to IMNCI guidelines, transferring complementary skills to parents required more attention [84]. Inadequate provider capacity could lead to inconsistent advice, misinformation, negative attitudes. This coupled with lack of time, and poor hospital policies may create barriers to successful implementation of the baby friendly breastfeeding initiative [24]. While training of providers is recognized as fundamental, few interventions emphasized provider attitudes and behavioral aspects that may affect their communication with parents.

## Discussion

This scoping review describes literature on experience of care for sick young children (24 months of age and younger) and the experiences of their parents or caregivers, the factors driving negative experience of care, and interventions to improve these experiences and address the emotional needs of parents with sick young children. The 68 published and grey literature documents assessed from the last 10 years reveal that findings on mistreatment of young children, positive experiences, and interventions in a hospital setting emerged primarily from Asia, Europe, or North America, while most literature on the drivers of mistreatment is from sub-Saharan Africa. Overall, the literature on experiences of care is limited; there is little on mistreatment of newborns and young children (particularly infants and young children of ages 60 days to 24 months) and even less on how to mitigate mistreatment broadly. Interventions to improve the experience of care—nurturing care, FCC, parental engagement, and provider communication—are generally implemented in high income settings, with a paucity of evidence on how such models can be implemented in low income settings, with a focus on small and sick newborns and young children with chronic illnesses or conditions. Existing models do not sufficiently distinguish how provider norms and attitudes toward parents affect their interactions and behaviors.

### Experience of care for sick young children

All children have a right to respectful and adequate health care that considers their behavioral and developmental needs, and is sensitive to the emotional and psychological needs of their caregivers [85–88]. Authors identified three sets of negative experiences: mistreatment of newborns; poor provider communication and its effect on parents and their children; and the negative effects of newborn and young child separation from their parents or families while in hospital. Most of these studies were in the United States and Europe.

### Drivers of negative care experience of care

This scoping review shows that the drivers of negative experience of care are well documented, especially for newborns (n = 10), but little evidence exists for young children up to 24 months (n = 1). Factors driving inadequate provider care include inadequate knowledge, poor communication and interaction with parents, and poor personal and professional attitudes. Factors affecting care at facilities include high workloads with inadequate staffing, commodities, equipment, and general infrastructure. These factors are well described in high income countries, but there is little recorded evidence on negative experiences of care especially in sub-Saharan Africa [89–91].

### Interventions to improve positive experience

This review reveals a wide range of interventions (n = 32) for improving quality of care, especially those involving families. These strategies are mainly articulated for small and sick newborns and premature infants including children with long term or chronic illnesses, with limited evidence on older children ages 60 days to 24 months.

This scoping review identified three inter-related frameworks that have been introduced in high income countries: 1) Family-Centered Care (FCC), 2) Family and Parent Engagement, and 3) Nurturing Care. The three frameworks put the child’s family at the center of childcare during hospitalization. Each emphasizes family education, coaching, and full partnership with health care providers for a child’s care, which is found to improve understanding and adherence to plan of care in addition to follow up care at home, resulting in better newborn and child health outcomes. Supporting parents psychosocially while at the hospital and continually once back in society–particularly in cases of perinatal loss remains an area for future adaptation and refinement. Good provider communication and counseling also enhance the experience of care with improved mutual understanding of care measures and enhanced respect among caregivers, families, and providers.

Factors that influence family engagement include beliefs about the family/of child’s role, health literacy and education, organizational culture, societal norms, policy, practice and regulation [59]. WHO standards for improving the quality of care for small and sick newborns in health facilities recommends adequate providers and capacity building of existing newborn management including nurturing care for sick newborn and their families through orientation programmes, continuing education, skills training, quality improvement initiatives and support to maintain and increase competence [92]. But there is minimal evidence on child health policies and protocols that guide implementation of these frameworks and interventions in resource-constrained settings. We also noted inadequate focus on provider norms, attitudes, and biases that may affect the quality of their communication and engagement with parents. Our results show that parental and provider knowledge, along with protocols and job aids and visual tools can positively or negatively influence the degree of willingness to engage in strategies for enhanced care for newborns and young children including for a critically ill infant.[36, 93–97].

Several studies show that a conducive policy environment, facility preparedness, and improved provider and parental knowledge play critical roles in successful implementation of health care interventions [93, 94, 96, 98]. One area of evidence that apparently mitigates poor experiences of care is the promotion of shared decisions by parents and providers in children’s care a key element of family centered care and parent engagement. While we did not include this in our search terms, a 2019 systematic review of “barriers to shared decision-making between parents and providers” identified that the type of decision, poor quality information, a caregiver’s emotional state, relative power relations, and inadequate time are key issues that need to be addressed [99].

### Strengths and limitations

This scoping review adds to the existing body of evidence on experience of care and the factors that drive mistreatment of newborn and young children up to 24 months of age. It also describes promising intervention models to improve the experience of care and reduce mistreatment. The process of interpreting evidence from the interventions in this review was complicated since many studies did not consistently nor adequately measure outcomes. Although the authors conducted an extensive in-depth literature review, it is possible not all available literature was identified, as only those studies published in English and within the last 10 years were included. Moreover, similar to other scoping reviews and in line with the debate around quality assessment constraints, the study did not assess quality of evidence to the extent possible in a in a systematic review with tighter criteria and sufficient research in the topic area [18, 100]. Despite these limitations, including the expanding and varied definitions of the methodological approach, this scoping review suggests a range of models that could be adapted and integrated within programs and policies for improving hospital-based experiences of care for newborns and young children as well as their parents; and can be introduced and piloted in low income settings.

This scoping review will be used in two ways: 1) with formative research in select hospitals in Kenya for developing a contextually-relevant model to improve experience of care for parents and families seeking services for sick young children up to 24 months in a low income setting, and 2) to develop and test a provider behavior change approach to enhance provider and parent communication and engagement for the promotion of respectful, nurturing and health system responsive care to newborns, infants, and young children in a low resource setting.

## Conclusion

Experience of care is an integral component of quality care for hospitalized newborns and young children up to 24 months of age. There is limited evidence on the implementation of models of care that sufficiently consider provider-centric norms and attitudes while involving parents and families in the care of hospitalized newborns and young children globally, and in sub-Saharan Africa. A focus on addressing norms and values of health providers that may drive poor quality of care is critical. Improving provider performance is crucial to accelerating progress in child survival—and to thrive—and meeting the sustainable development goal targets.

## Supporting information

S1 Checklist Prisma checklist. (TIF)

S1 File Pubmed full electronic search strategy. (ZIP)

S2 File Updated search strategy. (TIF)

S1 Table (TIF)

## Abbreviations

ENAP: Every Newborn Action Plan
FCC: Family Centered Care
IMNCI: Integrated Management of Newborn and Child Initiative
LMICs: Low and Middle-Income Countries
MNH: Maternal and Newborn Health
NICU: Neonatal Intensive Care Unit
WHO: World Health Organization
