# Supplementary figures and images for: Experience of care of hospitalized newborns and young children and their parents: A scoping review
Source: PLoS One. 2022 Aug 29;17(8):e0272912. doi: 10.1371/journal.pone.0272912 (PMC9423633; doi:10.1371/journal.pone.0272912)

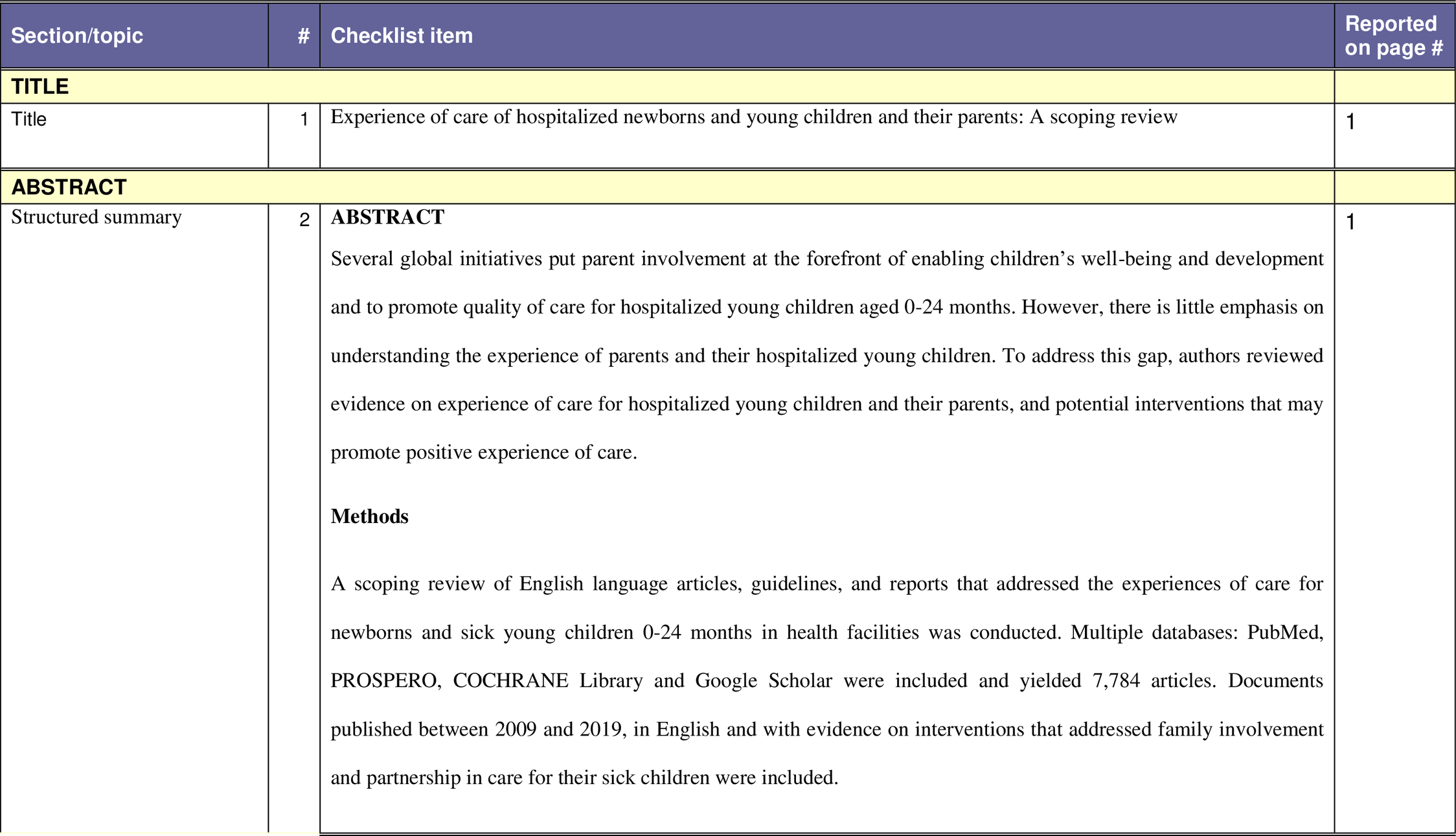

Supplement: S1 Checklist — (TIF) [file pone.0272912.s001.tif]

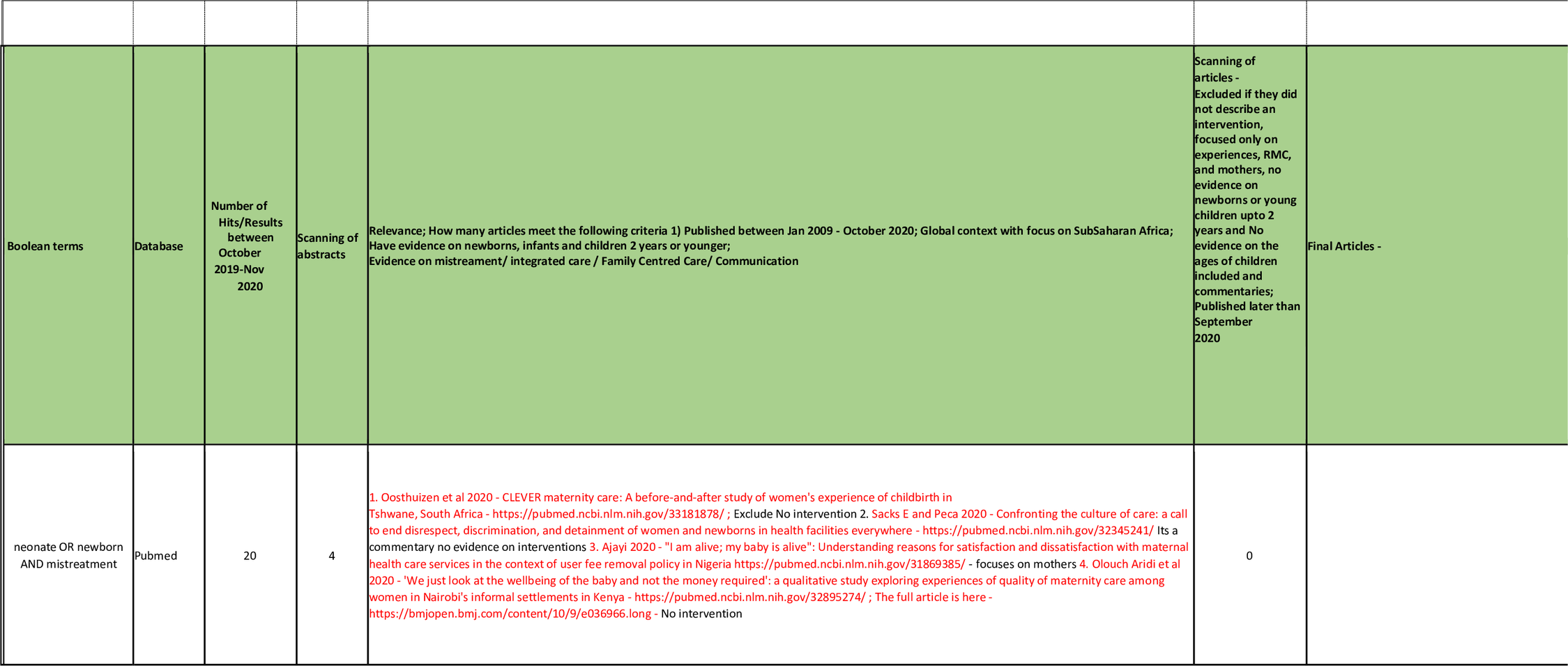

Supplement: S2 File — (TIF) [file pone.0272912.s003.tif]

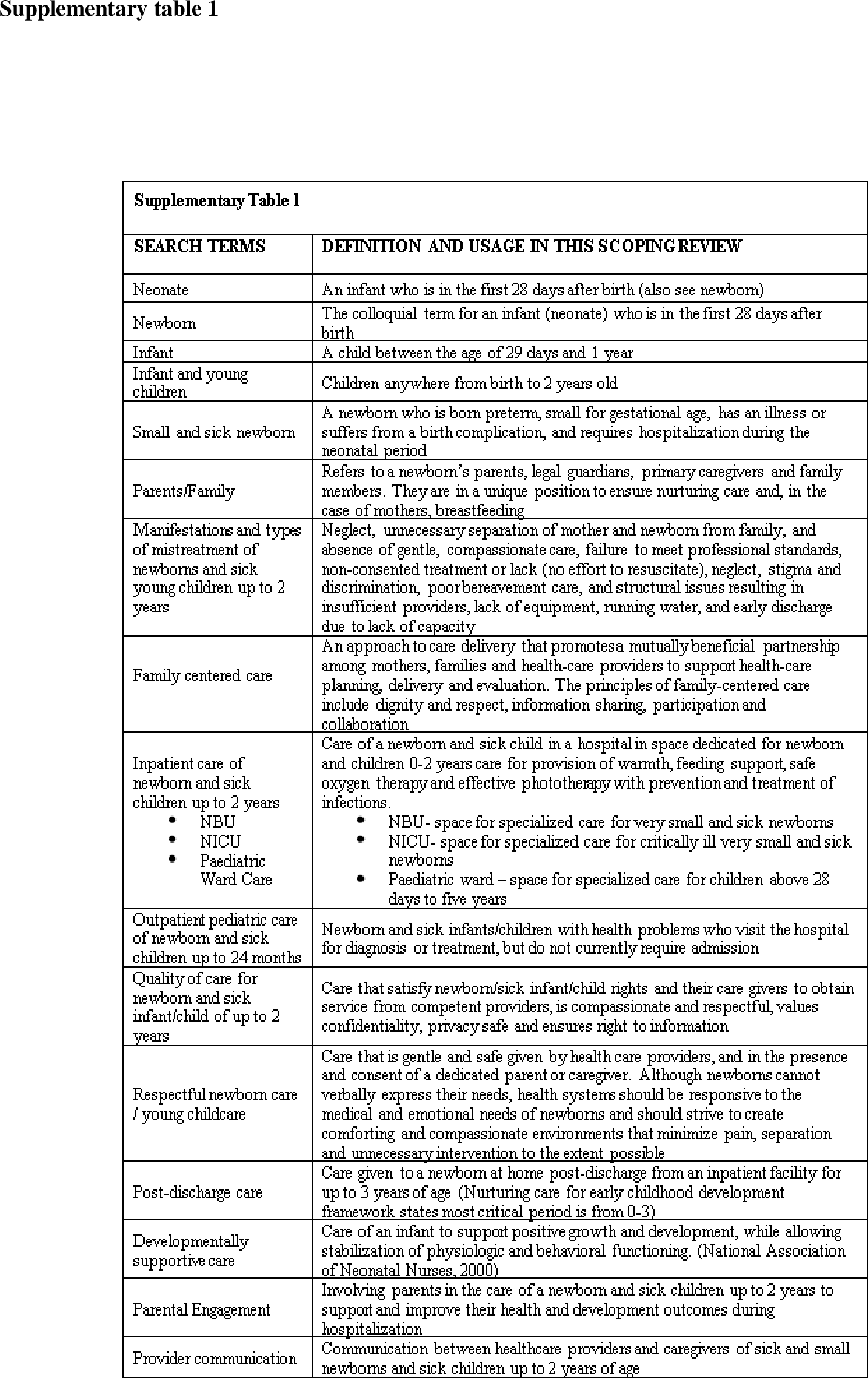

Supplement: S1 Table — (TIF) [file pone.0272912.s004.tif]
